# Endoscopic Outcomes Before and Five Years After Laparoscopic Sleeve Gastrectomy: Is There a Significant Impact?

**DOI:** 10.7759/cureus.70009

**Published:** 2024-09-23

**Authors:** Owaid M Almalki, Tamer M Abdelrahman, Mohammed E Mukhliss, Dhuha A Alhumaidi

**Affiliations:** 1 Department of Surgery, College of Medicine, Taif University, Taif, SAU; 2 Department of Surgery, Armed Forces Hospitals, Taif, SAU; 3 Department of Surgery, Benha Teaching Hospital, Benha, EGY; 4 Department of Internal Medicine/Gastroenterology, Alhada Armed Forces Hospital, Taif, SAU

**Keywords:** esophageal symptoms, long-term results, endoscopy, gastroesophageal reflux disease, laparoscopic sleeve gastrectomy

## Abstract

Laparoscopic sleeve gastrectomy (LSG) is a popular bariatric procedure with significant effects on weight and metabolic health. However, its impact on gastroesophageal reflux disease (GERD) and esophageal symptoms remains debated. This study aims to evaluate the endoscopic changes five years post-LSG. We conducted a retrospective analysis of patients who underwent LSG at our center between June 2017 and June 2019. Inclusion criteria included preoperative and at least five-year follow-up esophagogastroduodenoscopy (EGD). We analyzed demographic factors, esophageal symptoms, and endoscopic findings.

Out of 118 patients who underwent LSG, 24 met the inclusion criteria. Two patients were excluded due to conversion to Roux-en-Y gastric bypass (RYGB). The final cohort included 22 patients with a mean age of 42 ± 10 years and a mean BMI of 45 ± 7 kg/m². Preoperative EGD showed no GERD in 21 patients (95.5%) and GERD in 1 patient (4.5%). At five-year follow-up, 14 patients (63.6%) had no GERD, 7 (31.8%) had GERD A, and 1 (4.5%) had GERD B. Esophageal symptoms included heartburn (54.5%), nausea (36.4%), regurgitation (31.8%), and epigastric pain (22.7%). PPI or antacid use was reported in 10 patients (45.5%). Extra-esophageal symptoms were rare. BMI was significantly higher in patients with GERD (34.5 ± 6.3 kg/m²) compared to those without GERD (30.2 ± 5.1 kg/m², p = 0.04).

LSG may lead to the development or worsening of GERD in a subset of patients, despite the majority showing no significant GERD changes over five years. Continuous monitoring and tailored management strategies are essential for optimizing outcomes.

## Introduction

It is anticipated that the global obesity epidemic will reach a prevalence of 51% by 2030. Metabolic bariatric surgery (MBS) is the most effective intervention for severe obesity and its associated medical issues [1]. In recent years, laparoscopic sleeve gastrectomy (LSG) has increasingly been used as a primary bariatric procedure and has gained popularity among both patients and surgeons due to its effectiveness in achieving significant weight loss and resolving related illnesses [2]. It has also been utilized as a form of metabolic surgery because of its efficacy in altering anti-diabetic hormones [3].

Laparoscopic sleeve gastrectomy (LSG) is accomplished using a basic surgical method with minimal alterations to the native anatomy. It does not involve the use of foreign materials or the creation of gastrointestinal connections, and it maintains the natural functioning of the gastrointestinal system [4]. However, the impact of LSG on gastroesophageal reflux disease (GERD) remains uncertain, as there is inconsistent data on the management of pre-existing reflux and the development of new-onset GERD following the procedure [5].

GERD-related symptoms are consistently more prevalent in obese and morbidly obese populations compared to non-obese individuals. Some studies report an incidence of up to 50% in those with a BMI greater than 30 kg/m² [6]. Individuals with obesity are at a higher risk of reflux due to several physiological factors, including temporary relaxation of the lower esophageal sphincter, increased intra-abdominal pressure, a greater pressure differential between the stomach and esophagus, and a higher incidence of hiatal hernia [7].

Hampel et al. conducted a meta-analysis of nine studies, finding that individuals with a BMI of 25-30 kg/m² had a pooled adjusted odds ratio of 1.43 for GERD symptoms. For those with a BMI over 30 kg/m², the odds ratio was 1.94, highlighting a clear association between obesity and reflux, with the risk increasing as BMI increases [8].

Additionally, Trujillo et al. included 22 studies in their meta-analysis, involving 20,495 participants, and found that the estimated proportion of patients who developed post-surgery GERD was 0.35 (95% CI 0.30-0.41). Subgroup analysis showed a proportion of 0.33 (95% CI 0.27-0.38) in observational studies and 0.58 (95% CI 0.39-0.75) in clinical trials [9].

Multiple studies have assessed the impact of LSG on pre-existing GERD and the development of new-onset GERD, yielding conflicting findings. Some individuals reported improvements in their GERD [10-13], while other studies demonstrated the development of new cases of GERD after surgery or the worsening of pre-existing GERD [14-17].

The occurrence of de novo GERD following LSG might be attributed to anatomical changes and technical inaccuracies. After LSG, the stomach transitions from a wide, flexible shape to a long, narrow tube, leading to reduced compliance and increased intraluminal pressure.

However, the improvement of GERD after LSG can be explained by the reduction in abdominal pressure resulting from weight loss, decreased acid production due to fundus removal, and accelerated gastric emptying [12,13]. Furthermore, analysis of the angle between the His and the left crus muscle shows that pressure exerted by the esophageal sphincter, which is responsible for maintaining the anti-reflux mechanism, is reduced. Technical mistakes include angle narrowing, gastric tube twisting, anatomical stenosis, and the persistence of an undiagnosed hiatal hernia post-surgery [18].

The discrepancies in these findings emphasize the necessity for more research on the enduring consequences of LSG on GERD and its endoscopic symptoms. While there have been some studies investigating the connection between LSG and GERD, there is currently a lack of a thorough assessment of endoscopic observations after a five-year follow-up. The lack of research in this area impedes our comprehension of the lasting impacts on the esophageal surface caused by LSG and any potential enhancements or improvements in issues associated with GERD.

Aim

The objective of this study is to evaluate the endoscopic outcomes after five years in a group of patients who underwent LSG, with a particular emphasis on examining the relationship between GERD and esophagitis. Through the examination of endoscopic findings before and after surgery, our objective is to ascertain if there are substantial long-term alterations and to understand the consequences of these changes for patient care.

## Materials and methods

Study design

We conducted a prospective cohort study at the Alhada Military Hospital, Taif, Saudi Arabia, to assess the impact of LSG on GERD through endoscopic evaluations performed preoperatively and at a five-year follow-up.

Among the 118 patients who underwent LSG from June 2017 to June 2019, only 24 patients met our eligibility criteria. Of these, 22 patients successfully completed both preoperative and five-year follow-up endoscopies. Eligibility criteria included adult patients with severe obesity, no prior history of major upper gastrointestinal surgery, and complete clinical data. Patients with severe comorbidities, gastrointestinal cancer, or a hiatal hernia (as identified by preoperative endoscopy or discovered intraoperatively) were excluded.

Ethical considerations

The study was approved by the Institutional Review Board (IRB) of Alhada Military Hospital. Written informed consent was obtained from all participants prior to their inclusion in the study.

Data collection

Data collected included demographic information, preoperative BMI, and endoscopic findings. Follow-up visits involved clinical assessments and endoscopic evaluations at regular intervals: one month, three months, six months, one year, three years, and five years postoperatively.

The primary outcome measures were the presence of GERD symptoms after surgery, the incidence of new-onset GERD and esophagitis postoperatively, and changes in the presence and grade of esophagitis from preoperative to five-year postoperative endoscopy. Secondary outcome measures included the identification of other pathological findings on endoscopy.

Statistical analysis

Data were analyzed using IBM SPSS Statistics for Windows, Version 25 (Released 2017; IBM Corp., Armonk, New York). Descriptive statistics summarized the data, while paired t-tests compared preoperative and postoperative findings. A p-value of <0.05 was deemed statistically significant.

Surgical technique

All LSGs were conducted using a three-port approach, consisting of two 5-mm ports and one 12-mm port. The procedure utilized a sealing device (Ligasure™, Medtronic, Covidien, Inc., Dublin, Ireland). The greater curvature of the stomach including the posterior fundus was mobilized completely from the pylorus to the angle of His exposing the left crus of the diaphragm. 

The greater curvature of the stomach was transected by a linear stapler (ECHELON FLEX™ Powered Staplers, Ethicon Endo-Surgery, Inc., Cincinnati, Ohio) from the antrum (3 cm from pylorus) to the angle of His with 36 Fr calibration tube. There was no utilization of staple line reinforcement or buttressing. Once the specimen was retrieved, clips were placed on the bleeding points of the staple line. The use of intra-abdominal drainage was omitted.

## Results

Of the 118 patients who underwent LSG at our center between June 2017 and June 2019, 24 met the inclusion criteria, having both pre-operative and at least five-year follow-up esophagogastroduodenoscopy (EGD). Two patients were excluded from the study after requiring conversion to Roux-en-Y gastric bypass (RYGB) within the first postoperative year due to GERD symptoms and EGD findings. As a result, 22 patients were included in the final analysis.

The study cohort consisted predominantly of Saudi nationals (18 patients, 81.8%), with a minority of non-Saudi residents (4 patients, 18.2%). The majority were women (16 patients, 72.7%), and the mean age at the time of surgery was 42 ± 10 years. The mean BMI was 45 ± 7 kg/m², indicating severe obesity. Pre-operative endoscopy revealed that 21 patients (95.5%) did not have GERD, with only 1 patient (4.5%) diagnosed with GERD prior to surgery. The mean follow-up period was 60 ± 14 months, providing a substantial timeframe for evaluating long-term outcomes post-LSG.

During the follow-up, the most reported esophageal symptoms were heartburn (12 patients, 54.5%), nausea (8 patients, 36.4%), regurgitation (7 patients, 31.8%), and epigastric pain (5 patients, 22.7%). Additionally, 10 patients (45.5%) were using proton pump inhibitors (PPIs) or antacids. Extra-esophageal symptoms were infrequent, with only 1 patient (4.5%) reporting teeth erosion and 2 patients (9.1%) reporting chronic cough. Notably, no cases of pneumonia, chronic sinusitis, or otitis media were reported.

At the five-year follow-up, endoscopy findings revealed that 14 patients (63.6%) did not develop GERD. Of the 8 patients (36.4%) who did develop GERD, 7 (31.8%) were classified as GERD A, and 1 patient (4.5%) as GERD B. Importantly, there were no instances of severe GERD (C or D) (Table 1).

**Table 1:** Clinical and endoscopic characteristics at five-year follow-up. PPI: proton pump inhibitor, GERD: gastroesophageal reflux disease.

Characteristics	Symptom	N (%)
Esophageal symptoms	Heartburn	12 (54.5%)
	Regurgitation	7 (31.8%)
	Epigastric pain	5 (22.7%)
	Nausea	8 (36.4%)
	Sleep difficulties	3 (13.6%)
	PPI or antiacid	10 (45.5%)
Extra-esophageal symptoms	Teeth erosion	1 (4.5%)
	Chronic cough	2 (9.1%)
	Pneumonia	0 (0%)
	Chronic sinusitis	0 (0%)
	Otitis media	0 (0%)
Endoscopy	No GERD	14 (63.6%)
	GERD A	7 (31.8%)
	GERD B	1 (4.5%)
	GERD C, D	0 (0%)

Analysis of demographic factors revealed no significant differences in age or gender between patients with and without GERD (p > 0.05). However, BMI was significantly higher in patients with GERD (mean BMI = 34.5 ± 6.3 kg/m²) compared to those without GERD (mean BMI = 30.2 ± 5.1 kg/m², p = 0.04) (Table 2).

**Table 2:** Demographic factors and post-endoscopic GERD outcomes. GERD: gastroesophageal reflux disease.

Demographic Factor	No GERD (n = 14)	GERD (n = 8)	p-value
Age (mean ± SD, years)	41.8 ± 9.6	42.5 ± 10.8	0.82
Gender			
Male, n (%)	4 (28.6%)	2 (25.0%)	0.84
Female, n (%)	10 (71.4%)	6 (75.0%)	
BMI (mean ± SD, kg/m²)	30.2 ± 5.1	34.5 ± 6.3	0.04

Further examination of the relationship between esophageal symptoms and post-endoscopic GERD outcomes revealed no significant associations. Patients with heartburn, regurgitation, nausea, or epigastric pain prior to endoscopy were not more likely to develop GERD post-operatively (all p-values > 0.05). This suggests that these symptoms alone may not reliably predict post-operative GERD, emphasizing the need for comprehensive diagnostic approaches, including endoscopy (Table 3).

**Table 3:** Relationship between esophageal symptoms and post-endoscopic GERD outcomes. PPI: proton pump inhibitor, GERD: gastroesophageal reflux disease.

Symptoms		Post-endoscopy GERD		Total	P-value
		No	Yes		
Heartburn	No	6 (42.9%)	4 (50.0%)	10 (45.5%)	0.75
	Yes	8 (57.1%)	4 (50.0%)	12 (54.5%)	
Regurgitation	No	8 (57.1%)	7 (87.5%)	15 (68.2%)	0.12
	Yes	6 (42.9%)	1 (12.5%)	7 (31.8%)	
Epigastric pain	No	11 (78.6%)	6 (75.0%)	17 (77.3%)	0.85
	Yes	3 (21.4%)	2 (25.0%)	5 (22.7%)	
Nausea	No	10 (71.4%)	4 (50.0%)	14 (63.6%)	0.32
	Yes	4 (28.6%)	4 (50.0%)	8 (36.4%)	
Sleeping difficulties	No	12 (85.7%)	7 (87.5%)	19 (86.4%)	0.91
	Yes	2 (14.3%)	1 (12.5%)	3 (13.6%)	
Use of PPI or antacid	No	8 (57.1%)	4 (50.0%)	12 (54.5%)	0.75
	Yes	6 (42.9%)	4 (50.0%)	10 (45.5%)	

Our findings indicate that a higher post-operative BMI is significantly associated with the development of GERD, highlighting the importance of effective weight loss in mitigating GERD symptoms post-LSG. Conversely, the presence of esophageal symptoms did not significantly correlate with GERD outcomes, underscoring the complexity of GERD diagnosis and the limitations of relying solely on symptomatology.

## Discussion

Research investigating the correlation between sleeve gastrectomy and reflux has yielded inconclusive results, with both elevated and reduced rates of reflux found after the procedure. Possible factors that may lead to an increase in reflux include obesity, heightened intragastric pressure, and a greater pressure difference between the esophagus and stomach, which might result in the development of a hiatal hernia, frequently observed among individuals with obesity. However, sleeve gastrectomy and subsequent weight loss can mitigate reflux by reducing these factors. Additionally, decreased acid production and accelerated gastric emptying may help alleviate reflux. Conversely, factors that could increase reflux after gastrectomy include reduced gastric compliance, increased intragastric pressure, decreased lower esophageal sphincter resting pressure, persistent hiatal hernia, and narrowing of the gastric angle [10].

Our study adds valuable insights to the growing body of literature on the impact of LSG on GERD and esophageal symptoms. While previous research has reported varying incidences of GERD following LSG, our findings stand out due to the extended five-year follow-up period. Notably, while most patients did not develop significant GERD, a substantial proportion experienced new-onset GERD postoperatively. These results align with prior studies, reinforcing the importance of continued surveillance and proactive management of GERD in LSG patients. This highlights the need for tailored postoperative care to mitigate long-term complications and improve patient outcomes.

In our study, GERD diagnosis was achieved through a combination of objective and subjective methods: via upper endoscopy and symptom assessment. This contrasts with other studies that diagnose GERD solely based on questionnaires or self-reported symptoms, potentially leading to variability in reported incidence rates [19-21].

Our study found that 36.4% of patients developed GERD within five years post-LSG, with 31.8% classified as GERD A and 4.5% as GERD B. These findings are consistent with Dimbezel et al., who reported a significant prevalence of GERD five years after LSG based on endoscopic findings [22]. Similarly, Sebastianelli et al. highlighted a high rate of new-onset Barrett's esophagus following LSG, suggesting that LSG may predispose patients to significant esophageal changes over time [23]. However, two recent reviews by Altieri et al. and Nadaletto et al. concluded that the majority of studies associate LSG with worsening or “de novo” GERD after surgery, while improvement is only mentioned in a few papers [24,25].

Several mechanisms have been proposed to explain the development of new-onset GERD after LSG. The anatomical changes following LSG are key contributors. LSG, a restrictive procedure, creates a narrow gastric tube, altering the angle of His, which may predispose patients to GERD. Additionally, increased intragastric pressure, often due to variations in pouch shape, such as excessive narrowing or twisting, influenced by surgeon preferences and patient characteristics, can exacerbate GERD symptoms. Furthermore, potential damage to the sling fibers of the lower esophageal sphincter (LES) during stapling may lead to decreased basal pressure or transient relaxation of the LES, promoting GERD. Conversely, the reduction in parietal cell mass and rapid gastric emptying post-gastrectomy might provide some defense against GERD [25].

Numerous studies have emphasized the increased incidence of GERD following LSG, highlighting the long-term effects on esophageal health. Csendes et al. documented significant clinical, endoscopic, and histologic changes in the distal esophagus and stomach over a 10.5-year follow-up period, concluding that LSG appears to be a "pro-reflux" procedure that warrants ongoing evaluation long after surgery [26]. Eric et al. predicted that the postoperative incidence of GERD and esophagitis would be 18% and 25.4%, respectively, at 2 years and 32.1% and 85.0% at 5 years [27]. In contrast, Rebecchi et al. observed that in obese patients without preoperative GERD, the occurrence of “de novo” reflux was uncommon, suggesting LSG can be an effective option for obese patients with GERD [13].

Analysis of demographic factors as a risk factor for the development of GERD after LSG revealed no significant differences in age or gender between patients with and without GERD (p > 0.05). This is consistent with earlier studies that found no correlation between age and the development of GERD following LSG [21,28]. Conversely, a study revealed that older age was linked to a greater occurrence of GERD [29]. Our analysis also showed no significant differences in gender between patients with and without GERD (p > 0.05), consistent with the findings of Althuwaini et al. [21].

A significant association was found between higher postoperative BMI and the development of GERD, with patients who developed GERD presenting a mean BMI of 34.5 ± 6.3 kg/m² compared to 30.2 ± 5.1 kg/m² in those without GERD (p = 0.04). This underscores the importance of effective weight management in mitigating GERD post-LSG. As noted by Csendes et al., sustained weight loss post-LSG can reduce abdominal pressure and acid production, alleviating GERD symptoms [26]. This result is consistent with the findings of Angrisani et al., where the absence of GERD was linked to better weight loss outcomes at five years [3].

Interestingly, our study highlights that insufficient weight loss or weight regain is a significant predictor of GERD postoperatively. Patients who failed to achieve substantial weight reduction were more likely to develop GERD symptoms, emphasizing the need for sustained weight loss strategies to reduce the risk of reflux. These findings align with the work of Melissas et al., who similarly reported that weight regain after LSG was associated with a higher incidence of GERD, underlining the importance of sustained weight loss for long-term GERD management [30]. Further supporting these findings, Carter et al. demonstrated that patients who failed to achieve sufficient weight loss post-LSG were more likely to experience GERD symptoms, highlighting the necessity of effective postoperative weight management strategies [31].

Our study also found that esophageal symptoms such as heartburn, regurgitation, nausea, and epigastric pain did not significantly correlate with postoperative GERD outcomes. This underscores the complexity of GERD diagnosis and the limitations of relying solely on symptomatology. Accurate assessment and management of GERD post-LSG require comprehensive diagnostic approaches, including endoscopy and esophageal pH-metry.

The frequent use of PPIs or antacids in nearly half of the patients postoperatively underscores the pressing need for individualized pharmacologic interventions. A study involving 110 patients who underwent LSG reported a significant increase in PPI usage postoperatively compared to preoperative levels (57.2% vs. 19.1%, p < 0.0001) [32]. This sharp rise in PPI intake highlights the potential inadequacy of current management strategies and the necessity for tailored therapeutic approaches to effectively address the elevated risk of GERD and related symptoms following LSG.

As Benvenga et al. concluded, the frequency and severity of endoscopic anomalies observed with an average follow-up of more than six years call for a policy of systematic upper endoscopies for long-term control after LSG [33]. Similarly, Sebastianelli et al. noted that systematic endoscopic follow-up is crucial for the early detection and management of esophageal complications, such as Barrett's esophagus, which has significant implications for long-term patient care [23]. These findings collectively suggest that a more tailored approach to both pharmacologic and endoscopic management is essential to optimize patient outcomes and mitigate the risk of serious esophageal complications.

The results of this study suggest that patients undergoing LSG should be counseled on the potential risk of developing GERD and the importance of long-term follow-up. Tailored management strategies, including routine endoscopic surveillance and weight management programs, may be essential in mitigating these risks. Furthermore, surgical techniques that minimize intraluminal pressure and prevent the formation of hiatal hernias could potentially reduce the incidence of GERD postoperatively.

Limitations

Although this study has provided helpful insights, it is crucial to recognize certain limitations. The limited sample size may restrict the applicability of our findings. Despite its informative and useful nature in GERD patients, the implementation of esophageal pH-metry was not available in this cohort.

To strengthen the validity of our results, future studies should involve larger cohorts and diverse populations. Additionally, our study focuses on the short- to mid-term effects of LSG; therefore, further research is needed to assess the long-term impact on GERD. Future investigations should also explore how variations in surgical techniques and modifications could mitigate the risk of postoperative GERD.

## Conclusions

In conclusion, while LSG remains a popular and effective bariatric procedure, our findings underscore the importance of continuous monitoring and tailored management strategies to optimize patient outcomes post-LSG. Effective weight management and comprehensive diagnostic approaches, including routine endoscopic evaluations, are critical in mitigating the risk of GERD. Ongoing research is essential to enhance our understanding of the complex relationship between LSG and GERD and to develop evidence-based guidelines for managing these patients.
